# OLIGOPELVIS – GETUG P07: a multicentre phase II trial of combined salvage radiotherapy and hormone therapy in oligometastatic pelvic node relapses of prostate cancer

**DOI:** 10.1186/s12885-015-1579-0

**Published:** 2015-09-25

**Authors:** Stephane Supiot, Emmanuel Rio, Valérie Pacteau, Marie-Hélène Mauboussin, Loïc Campion, François Pein

**Affiliations:** 1 Department of Radiation Oncology, Institut de Cancérologie de l’Ouest, Boulevard J. Monod, F-44800 Nantes, St-Herblain France; 2 Centre de Recherche en Cancérologie Nantes-Angers (CRCNA), UMR 892 Inserm - 6299 CNRS, Institut de Recherche en Santé de l’Université de Nantes, 8 quai Moncousu, BP 70721, 44007 Nantes cedex 1, France; 3 Department of Research, Institut de Cancérologie de l’Ouest-René Gauducheau, boulevard Jacques Monod, Saint Herblain, 44800 France; 4 Department of Statistics, Institut de Cancérologie de l’Ouest-René Gauducheau, boulevard Jacques Monod, Saint Herblain, 44800 France

## Abstract

**Background:**

Metastatic prostate cancer remains a common cause of death in Europe, and improvements in management of the disease are urgently needed. The advent of positron-emission tomography (PET) imaging enhanced with fluorocholine has led to the identification of a new sub-group of metastatic prostate cancer patients: those with so-called oligometastatic disease. Presenting with a low burden of metastatic disease (≤5 lesions), this new sub-group lies between true metastatic prostate cancer patients for whom androgen- deprivation therapy (ADT) is the mainstay of treatment, and patients with a rising PSA, but no visible lesion on conventional imaging, in whom intermittent ADT has been shown to be no less effective than continuous ADT. One might conclude that intermittent ADT would also be the standard of care for oligometastatic prostate cancer patients, but radical strategies such as extensive lymphadenectomy or high-dose radiotherapy have been suggested as another means to delay the need for ADT, and increase its effectiveness once initiated. This study will explore the role of salvage pelvic image-guided intensity-modulated radiation therapy (IMRT) combined with ADT administered for 6 months in pelvic oligometastatic patients in prolonging the failure-free interval between two consecutive ADT courses, or even to cure selected patients with limited metastatic burden.

**Methods/Design:**

We plan to assess the two year outcome in oligometastatic prostate cancer patients (1–5 pelvic oligometastases) treated concomitantly with high-dose IMRT (54 Gy, 30 fractions to the pelvis and 66 Gy, 30 fractions to the lymph nodes) and ADT for six months.

**Discussion:**

This multicenter prospective phase II study will yield new data regarding the safety and efficacy of high-dose radiotherapy combined with ADT and will provide a basis for a larger phase III study to examine the role of radiotherapy in this population currently treated only with hormone therapy.

**Trial registration:**

NCT02274779, date of registration: 23/10/14

## Background

Improvements in the management of metastatic prostate cancer are urgently needed given the high number of prostate cancer deaths in Europe. The advent of FCH-PET imaging has recently led to identification of more prostate cancer patients now termed oligometastatic (five or fewer lesions) who would otherwise be considered clinically negative using whole body bone scanning and CT [1]. Oligometastatic disease has also been recognized in other tumor types such as colon, lung, or breast cancers, and has influenced their management, with more radical treatments such as surgical resection or radical radiotherapy being employed [2].

**Fig. 1:**
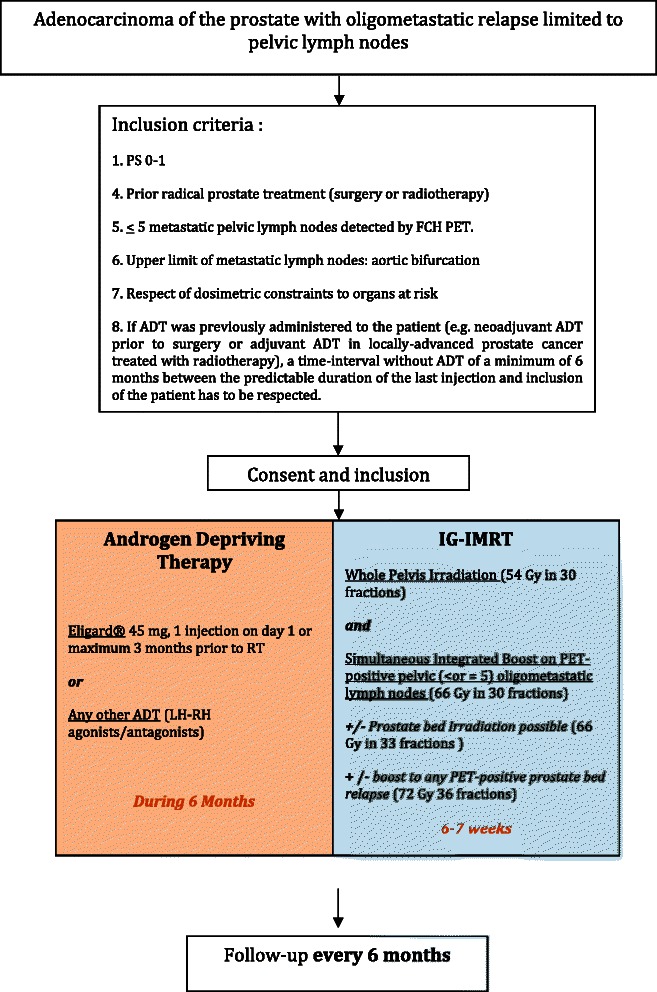
Study design

Continuous Androgen deprivation therapy (ADT) is recommended in metastatic prostate cancer patients [3]. At the opposite, the European Association of Urology states that no ADT is recommended in patients with a rising PSA but no visible lesion on conventional imaging (http://uroweb.org/individual-guidelines/oncology-guidelines). The group of oligometastatic prostate cancer patients clearly falls between these two categories and intermittent ADT could represent a practical option for oligometastatic patients [4, 5].”

Radical strategies such as extensive lymphadenectomy or high-dose radiotherapy have been explored in the quest to improve the efficacy of ADT or delay its initiation [6]. Extended lymph node dissection is a good option for selected patients: one single institution series showed an encouraging rate of long-term biochemical control of 29 % at 5 years among patients demonstrating an initial response [7]. Similar results have been obtained using radiotherapy, with a limited number of mostly single-institution retrospective studies reporting good biochemical control in a majority of patients, with 30-month progression-free survival rates of more than 40 %, with some, low, toxicity [8, 9]. Overall, 51 % of patients are progression free one to three years after salvage surgery or radiotherapy in oligometastatic prostate cancer patients with disease confined to the pelvis [10].

Different radiotherapy strategies are possible. High-dose stereotactic body radiotherapy (SBRT) offers the potential of irradiating each individual metastatic lymph node using with a limited number of fractions and is well-tolerated overall [2]. It is also possible to irradiate all pelvic lymph nodes with high doses [11] combined with a boost to PET-positive lymph nodes using image-guided, intensity-modulated radiation therapy (IG-IMRT). The choice between the two strategies is influenced by surgical series that suggest that FCH-PET may miss micrometastatic disease in neighboring lymph nodes [12]. We have therefore favoured IG-IMRT, treating all of the pelvic lymph nodes, with a boost to PET-positive lymph nodes. Prostate cancer patients may relapse in pelvic lymph nodes as well as in retroperitoneal lymph nodes up to the level of L2-L3 [13]. However, to avoid potentially increased bowel toxicity, the Groupe d’Etude des Tumeurs Uro-Génitales (GETUG) committee recommends setting the upper limit of the radiation field to the level of the bifurcation of the aorta.

There is a limited knowledge about the importance of aggressive salvage treatments of oligometastatic prostate cancer [14]. Most studies are single-institution retrospective studies. This study will be the first prospective multicenter study of a homogenous patient population treated with high-dose IG-IMRT. Other clinical trials involving oligometastatic prostate cancer patients are currently exploring different options:

- NCT01859221: A single-institution phase II stereotactic body radiotherapy and stereotactic hypofractionated radiotherapy for oligometastatic hormone sensitive and hormone-resistant prostate cancer
- NCT0219278: SBRT as a treatment of oligometastases of prostate cancer
- NCT01558427: a randomized Phase II Trial comparing salvage treatment (surgery or SBRT) or active clinical surveillance for oligometastatic prostate cancer [15]

In summary, the literature suggests that oligometastatic prostate cancer patients may be successfully treated with intermittent ADT and high-dose irradiation to the metastatic lesions. The GETUG strategy is now to treat these metastatic patients with curative intent rather than with palliative hormone therapy as currently. This strategy aims to prolong the failure-free interval between two consecutive ADT courses, or even cure selected patients with limited metastatic burden. In this phase II study we plan to assess the two-year outcomes of oligometastatic prostate cancer patient (1–5 pelvic oligometastases) treated concomitantly with high-dose IMRT and 6-month LH-RH agonists.

## Methods/Design

### Hypothesis

We hypothesize that salvage pelvic lymph node IG-IMRT combined with 6-month ADT will achieve a 2-year relapse-free survival of 70 % in hormone-sensitive prostate cancer patients. Study design is described in Fig. 1.

### Study objectives

The main objective is to assess biochemical-clinical failure: defined by a cluster of events including PSA progression (defined below) or clinical evidence of local (defined below) or metastatic progression (defined below), or post-treatment initiation of hormonal therapy by the treating physician, or prostate cancer-related death.

The initial PSA prior to initiation of ADT will be the starting point. PSA progression is defined by

- In case of decline from starting point: record time from randomization to first PSA increase that is ≥25 % and ≥ 2 ng/ml above the nadir OR that is ≥25 % and rises above the pre-treatment PSA value and which is confirmed by a second value 3 or more weeks later.
- In case of no decline from starting point: PSA increase that is ≥25 % and ≥ 2 ng/ml after 3 months if baseline PSA is ≥2 ng/ml. PSA increase that is ≥25 % after 3 months if starting point PSA is < 2 ng/ml.

Local progression of pelvic FCH PET-positive lymphnodes is defined using the RECIST criteria [16].

Metastatic progression is defined as the appearance of new metastatic lesions.

Secondary objectives include:

- overall survival
- acute and late toxicity: evaluated by CTC-AE v4
- quality of life: EORTC QLQ-C30 and PR25 questionnaires
- site of tumor progression: using fluorocholine PET at biochemical relapse
- Time to start of palliative ADT: ADT will be started at time of polymetastatic disease, local progression or symptoms.

### Main inclusion criteria

1. Prostate adenocarcinoma;
2. ≥ 18 years
3. Performance Status 0–1
4. Prior radical prostate treatment (surgery or radiotherapy)
5. ≤ 5 metastatic pelvic lymph nodes detected by FCH PET.
6. Upper limit of metastatic lymph nodes: aortic bifurcation
7. Respect of dosimetric constraints to organs at risk
8. If ADT has been previously administered to the patient (e.g. neoadjuvant ADT prior to surgery or adjuvant ADT in locally-advanced prostate cancer treated with radiotherapy), a minimum of 6 months must have elapsed between the predicted duration of the last injection and inclusion of the patient in the study. For this category of patients, serum testosterone has to be higher than 6 nmol/l prior to inclusion.
9. Biochemical relapse defined according to the European Association of Urology guidelines [17]

### Main exclusion criteria

1. Bone or visceral metastases
2. Para-aortic lymph node metastases (above the aortic bifurcation)
3. Presence of more than 5 metastatic lymph nodes
4. Evidence of metastases at initial diagnosis
5. Evidence of distant metastases beyond the pelvic lymph nodes or prostate bed
6. Previous irradiation of pelvic lymph nodes
7. Castration-resistant prostate cancer as defined by the EAU guidelines [17]
8. Contraindications to pelvic irradiation (e.g. chronic inflammatory bowel disease)
9. Contraindications to ADT
10. Severe uncontrolled hypertension ( ≥160 mm Hg systolic and/or ≥ 90 mm Hg diastolic).
11. Other concomitant cancer or history of cancer (within the five years prior to study entry), except basal or squamous cell carcinoma of the skin provided they have been clinically inactive for at least five years.
12. Patients with a biochemical relapse while on active treatment with LHRH-agonist, LHRH-antagonist, anti-androgen, maximal androgen blockade, oestrogen.
13. Treatment during the past month with products known to influence PSA levels
14. (e.g.fluconazole, finasteride, corticosteroids…)
15. Disorder precluding understanding of trial information or informed consent
16. In case of previous prostate bed radiotherapy, FCH PET-positive lymphnodes have to be at least 1 cm above the upper limit of the irradiation field of the prostate bed to avoid overdosage.

## Statistics

A one-step Phase 2 Fleming design has been chosen. In addition, an analysis of acute toxicity persisting at one month after radiotherapy will be performed after the inclusion of the 20th patient to ensure that the toxicity of the irradiation protocol is not higher than expected.

### Statistical justification for the number of inclusions

It is difficult to accurately identify a control population. The recent adoption of FCH PET has enabled the detection of metastases in patients in biochemical relapse which the usual imaging modalities (bone scan, CT chest, abdomen and pelvis, pelvic MRI) were unable to detect. These patients therefore represent an intermediate group between patients with a rising PSA alone, and frank metastatic disease. To calculate the patient population, we can only rely on studies relating to a rising PSA patient population on one hand, and on first-line metastatic patients on the other hand. Prospective randomized data gathered from patients with a rising PSA showed that the median duration of biochemical control was 20 months with intermittent ADT [1]. Prospective randomized data concerning intermittent ADT in metastatic patients showed that 75 % of patients had resumed ADT after a mean duration of 15 months off [3]. Based on retrospective data, Ost et al. estimated that 51 % of patients are progression-free one to three years after salvage surgery or radiotherapy for pelvic oligometastatic disease [10]. Moreover, Rischke et al. showed a 50 % clinical recurrence at 2 year for patients undergoing both salvage lymphnode dissection and pelvic radiotherapy [18]. We therefore estimate that a biochemical relapse-free survival at 2 years of 70 % would be a significant result.

Based on a one-step Fleming design [19], we wish to be 95 % (α < 5 %) certain that any difference observed between the standard and experiment treatment groups is not due to the play of chance. We also want to be able to detect such a difference with 95 % power (b = 5 %). With these α and b, rejecting the null hypothesis H0: Π ≤ 50 %, and accepting the alternative hypothesis H1: Π ≥ 70 %, will require 63 evaluable patients.

Around 10 % of patients may be lost to follow-up before the evaluation of biochemical relapse at 2 years, so to ensure that we retain at least 63 patients until this evaluation it will be necessary to recruit 70 patients to this study.

If 39 or more of these 63 evaluable patients are still free of biochemical relapse at 24 months, we may reject the null hypothesis H0: Π ≤ 50 % with a risk of error of 3.85 % and a power of 93.5 % to detect the alternative hypothesis H1: Π ≥ 70 %.

### Level of statistical significance set

The power (1 - β) is 93.5 % and the α risk <5 %.

### Statistical criteria for stopping the study

If, among the first 20 patients, 10 or more patients present with grade 2 toxicity, we can state that the rate of grade 2 complications is greater than the reference norm of 30 % (p = 0.048). In this case the Data Safety Monitoring Board (DSMB) will meet to analyze the toxicity and decide whether to continue the study.

If, among the first 20 patients, 2 or more patients present with grade 3 toxicity, we can state that the rate of grade 3 complications is above the reference norm of 2 % (p = 0.059). In this case the DSMB will meet to analyze the toxicity and decide whether to continue the study.

## Detailed description of techniques to be used

1. *Androgen Deprivation Therapy (ADT)*
  The recommended ADT is Eligard ® 45 mg acting for 6 months. The use of other ADTs (LH-RH agonists or antagonists) is possible. The overall treatment duration is 6 months. It will be ideally administered on the first day of radiotherapy, or within the 3 months prior to the first day of radiotherapy.
2. *Radiation Therapy*

- General recommendations
  - The use of Intensity-Modulated Radiation Therapy (IMRT) is mandatory.
  - Irradiation will be delivered with a non-empty bladder and an empty rectum. It is recommended that the patient empties his bladder and rectum one hour before the planning CT-scan and before each radiotherapy treatment, and then drink at least 1/3 L of water after voiding. The one hour delay will be adapted to the patient’s continence.
  - Daily verification of patient’s organ positioning using IGRT is mandatory
  - Verification of *in vivo* dose delivery is mandatory
  - The use of a record and verify processing software is mandatory.
- Preparation of treatment
  - Planning CT of the pelvic cavity that includes all of the pelvic lymph nodes will be performed in the supine position, with *iv* injection of contrast agent to help visualize the pelvic vessels. Oral contrast agent will be administered to assist in the the determination of the small bowel volume. The minimum CT resolution will be at least 3 mm thick slices every 3 mm, but thinner slices may be necessary for very small volume lymphadenopathy. Merging the CT with pelvic MRI may help visualize the vascular axes and pelvic lymph nodes.
  - Fusion with FCH PET:
    The FCH PET images will be segmented using a threshold adapted to the size of the lymphadenopathy. These images will be used to locate the pathological lymph nodes, but the tumor volume (GTV) will be contoured on the planning CT.
  - Target volumes
  - Metastatic lymph nodes

GTV1 to GTV5 will be delineated on the planning CT-scan with the aid of FCH PET.

A margin of 5 mm around the GTVs will define the clinical target volumes (CTVs).

An additional margin of 5 mm around the CTVs will define the Planned Target Volumes (PTVs). The intersection between PTVs and small intestine will be removed from the PTVs.

- Whole pelvic lymph node irradiation

Pelvic lymph nodes will be contoured according to the recommendations in the RTOG consensus statement [20]. The pelvic lymph node CTV (PLN CTV) will include the common iliac, external iliac, internal iliac, presacral (S1-S3) and obturator regions. The PLN CTV is based on the pelvic vessels (arteries and veins) with an expansion of 7mm, reduced where necessary at the bone, muscle, bowel and bladder boundaries. The upper limit is defined by the bifurcation of the abdominal aorta. The lower limit will be at least 1 cm above the upper limit of the irradiation field of the prostate bed if this treatment has been performed previously.

A margin of 5 mm around the PLN CTV will define the PTV. The intersection between PTVs and the small bowel will be removed from the PLN PTV.

- Prostate bed

Irradiation of the prostate bed will be considered if the prostate bed has not been irradiated, or if a relapse is detected concurrently within the prostate bed.

The prostate bed CTV (PB CTV) will be defined according to the recommendations of the RTOG consensus [21].

- Boundaries

Below the upper edge of the pubic symphysis, its boundaries are:

- anteriorly: the posterior border of the pubic bone;
- posteriorly: the rectal wall;
- caudally: 8–12 mm below the vesicoureteral anastomosis;
- laterally: the levator ani and internal obturator muscle.

Above the upper edge of the pubic symphysis, its boundaries are:

- anteriorly: including 1–2 cm of the posterior wall of the bladder;
- posteriorly: the mesorectal fascia;
- cranially: the end of the vas deferens or 3–4 cm above the pubic symphysis;
- laterally: the sacro-recto-genito-pubic fascia.

A margin of 7 mm will be added to the PB CTV to form the PB PTV.

- Local relapse in the prostate bed (PBR GTV)
  It is possible to define a PBR GTV that encompasses the FCH-PET signal in the prostate bed, possibly with the aid of pelvic MRI. RPB CTV will be defined by a 5mm-margin around the PBR GTV. The PBR PTV will include the CTV with an extra margin of 5 mm.
- Organs at risk (OARs)
  OARs will be contoured as recommended by the RTOG [20]. It is not intended to add margins (PRV) around organs at risk.

- Bladder: the outer contour of the bladder on all sections where it appears. The bladder volume is the volume between the outer contour and an inner contour that is defined 7mm from the outer contour.
- Rectum: the external contour of the rectum from the recto-sigmoid junction to the anal canal. The rectal wall is the volume between the outer contour and an inner contour that is defined 5mm from the outer contour.
- Intestine: the external contours of the sigmoid, colon and the small intestine are defined as an “intestine bag” including the abdominal cavity as a whole. It will be important not to create areas of overlap between the intestine and the CTVs.
- Femoral head.
- Anal canal: 3 cm from its margin.

Dose prescription

- PTV1-PTV5: 66 Gy in 30 fractions of 2.2 Gy
- PLN PTV: 54 Gy in 30 fractions of 1.8 Gy
- PB PTV: 60 Gy in 30 fractions of 2 Gy. An additional 6 Gy (3x2 Gy fractions) can be delivered on the entire prostate bed.
- PBR PTV: 72 Gy in 36 fractions of 2 Gy (66 Gy to the PTV prostate bed plus an additional boost of 6 Gy -3x2 Gy)

Dose constraints

95 % of the PTV volumes will receive 95 % of the prescribed dose.

The QUANTEC recommendations advise that the maximum dose for the various organs at risk are:

- Bladder: the bladder volume receiving a dose > 65 Gy will be no greater than 50 %.
- Rectum: V50 < 50 %, V60 < 35 %, V65 < 25 %, V70 < 20 %, and V75 < 15 %.
- Bowel: the volume receiving 45 Gy will be less than 195 ml.
- Femoral head: <5 % of each femoral head should receive a dose > 50 Gy.
- Anal canal: D55 < 100 %.

Where the PTV coverage conflicts with the OAR constraints, the OAR protection will be privileged. For this reason, the PTV may be reduced to the CTV.

- Image guidance

Image-guided radiotherapy (IGRT) will be performed daily. The daily registration will be based at least on bone structures. It will be possible to readjust to the nodal structures in case of displacements > 1 cm.

- Quality control.

- Quality control of target volumes and critical organs

A GETUG RT quality assurance committee has been established consisting of a radiation oncologist and a radiation physicist. This committee is available for consultation should questions arise concerning the treatment protocol. The committee will initially accredit each centre on the basis of an electronic copy of a single case plan that demonstrates:

- CTV and PTV contoured according to protocol;
- organs at risk (bladder and rectal walls and femoral heads) contoured according to protocol;
- DVH for intestine, bladder and rectal wall, PTV and CTV that meet dose constraints;
- a statement that patients will be treated with an approved daily image guidance technique.

All treatment plans will be sent via a web-based platform for central reviewing and approval prior to the beginning of the radiotherapy treatment.

- Quality control of IMRT
  All IMRT plans will undergo quality assurance evaluation with ion chamber measurements or an equivalent method of dose verification to verify the absolute dose for each IMRT field and film dosimetry to measure the relative dose for each IMRT field, as in standard clinical practice. Independent Monitor Units calculation may be substituted for ion chamber dosimetry when available.

### Follow-up

Follow-up is described in Table 1. Quality of life will be evaluated prior to treatment and one month after completion of radiotherapy, and then every 6 months for 2 years using EORTC QLQ C30 and PR25. PSA and testosterone will be determined prior to radiotherapy, then 1 month after completion and then every 6 months. Repeat FCH-PET will be performed at biochemical recurrence.

**Table 1:** Study calendar

Visits	Inclusion	Treatment period	follow-up			
Visits	Max 2 months prior to RT day 1		M1 1 month after end of RT	Before progression		After progression
				Every 6 months during 2 years	Every 6 months until progression	Every 6 months until death
Inclusion/exclusion criteria	X					
Signed consent form	X					
Inclusion (max 2 months prior to RT)	X					
FCH PET	X (a)					X (b)
Physical examination with PS	X	X (d)	X	X	X	X
Prior history, tumor characteristics	X					
Blood Pressure	X	X (d)	X	X		
Acute toxicity during ADT and RT (f)		X (d)	X			
Late toxicity (f)				X		
QLQ-C30 et QLQ-PR25	X		X	X		
PSA	X		X (f)	X (f)	X (f)	
Testosterone	X (g)		X	X	X	
ADT (c) + RT		X				

(a) maximum 3 months prior to initiation of ADT

(b) at progression

(c) 6 months treatment staring on RT day 1 or maximum 3 months prior to RT

(d) once per week during RT

(e) biochemical progression = PSA higher than inclusion PSA with 2 successive rises in the same laboratory

(f) NCI-CTCAE v4.0

(g) 28 days prior to ADT

## Discussion

This trial addresses the use of radiotherapy and androgen deprivation therapy (ADT) in that emerging category of patients whose oligometastatic disease has been identified on fluorocholine PET imaging. This new category falls between true metastatic disease where ADT is a mainstay, and those patients with a rising PSA without visible lesions on conventional imaging, in whom intermittent ADT has been shown to be no less effective than continuous ADT. In this patient population, the role of radiotherapy is undetermined and two different approaches are currently in progess. The first approach is to consider that radiotherapy may delay the need for ADT by treating each visible oligometastasis using SBRT. Many single-center studies have been published (for review: [6]) and a randomized phase II study is comparing surveillance and salvage stereotactic radiotherapy or surgery to PET Choline-positive lymph nodes [15]. The second approach is that salvage radiotherapy may increase relapse-free survival or even cure some patients as based on recent retrospective series [9, 22]. Choline PET is currently the most validated prostate cancer staging modality, as compared to pelvic MRI, total bone scan or CT-scan. Other imaging modalities might perform better in the near future, such as whole body MRI or PSMA targeting radiolabeled agents. These imaging modalities are likely to become standard of care very soon and may further better define oligometastatic burden in future clinical trials. We feel that it is important to explore different approaches to treatment to inform the oncologists and urologists who are now routinely faced with the decision as to the best course of action in this new category of patients.

- Project planning:
  - The role of each team

It is expected that each team will recruit a mean number of 4 patients during the inclusion period. Each team will consist of a local radiation oncologist and his or her nuclear oncology and urology colleagues.

- Project calendar
  The study has been submitted and approved by regulatory authorities (ANSM; date of approval: 13/06/14) and ethics committee (Centre de Protection des Personnes Ouest IV - Nantes; date of approval: 3/05/14). The study opened in september 2014. A written informed consent will be obtained from the study participants. Recruitment is planned until the end of 2016. After a two-year minimum follow-up, final results will be presented during the last trimester 2018.
- Project co-ordination
  The project has been extensively discussed and approved by the GETUG. Patient recruitment will be monitored at each quarterly GETUG meeting.

## Abbreviations

ADT: Androgen deprivation therapy
PET: Positron emission tomography
FCH: ^18^F-Choline, fluorocholine
SBRT: Stereotactic body radiotherapy
IG-IMRT: Image guided-intensity modulated radiation therapy
GETUG: Groupe d’Etude des Tumeurs Uro-Génitales
PLN CTV: Pelvic lymph node clinical target volume
PB CTV: Prostate bed CTV
PBR GTV: Prostate bed relapse gross tumor volume
